# A single-source nosocomial outbreak of *Aspergillus flavus* uncovered by genotyping

**DOI:** 10.1128/spectrum.00273-24

**Published:** 2024-06-18

**Authors:** A. Gewecke, R. Krøger Hare, C. Salgård, L. Kyndi, M. Høg, G. Petersen, D. Nahimana, N. Abou-Chakra, J. D. Knudsen, S. Rosendahl, N. H. Vissing, M. C. Arendrup

**Affiliations:** 1 Mycology Unit, Department for Bacteria, Parasites, and Fungi, Statens Serum Institut, Copenhagen, Denmark; 2 Department for Clinical Microbiology, Rigshospitalet, Copenhagen, Denmark; 3 Department of Pediatrics and Adolescent Medicine, Copenhagen University Hospital, Rigshospitalet, Copenhagen, Denmark; 4 Section for Ecology and Evolution, Department for Biology, University of Copenhagen, Copenhagen, Denmark; 5 Department for Clinical Medicine, University of Copenhagen, Copenhagen, Denmark; Mayo Foundation for Medical Education and Research, Rochester, Minnesota, USA

**Keywords:** molecular epidemiology, genotyping, *Aspergillus flavus*, outbreak, hematologic malignancies, fungal air contamination, invasive aspergillosis

## Abstract

During construction work (2017–2019), an increase in *Aspergillus flavus* infections was noted among pediatric patients, the majority of whom were receiving amphotericin B prophylaxis. Microsatellite genotyping was used to characterize the outbreak. A total of 153 *A*. *flavus* isolates of clinical and environmental origin were included. Clinical isolates included 140 from 119 patients. Eight patients were outbreak-related patients, whereas 111 were outbreak-unrelated patients from Danish hospitals (1994–2023). We further included four control strains. Nine *A. flavus* isolates were from subsequent air sampling in the outbreak ward (2022–2023). Typing followed Rudramurthy et al.(S. M. Rudramurthy, H. A. de Valk, A. Chakrabarti, J. Meis, and C. H. W. Klaassen, PLoS One 6:e16086, 2011, https://doi.org/10.1371/journal.pone.0016086). Minimum spanning tree (MST) and discriminant analysis of principal components (DAPC) were used for cluster analysis. DAPC analysis placed all 153 isolates in five clusters. Microsatellite marker pattern was clearly distinct for one cluster compared to the others. The same cluster was observed in an MST. This cluster included all outbreak isolates, air-sample isolates, and additional patient isolates from the outbreak hospital, previously undisclosed as outbreak related. The highest air prevalence of *A. flavus* was found in two technical risers of the outbreak ward, which were then sealed. Follow-up air samples were negative for *A. flavus*. Microsatellite typing defined the outbreak as nosocomial and facilitated the identification of an in-hospital source. Six months of follow-up air sampling was without *A. flavus*. Outbreak-related/non-related isolates were easily distinguished with DAPC and MST, as the outbreak clone’s distinct marker pattern was delineated in both statistical analyses. Thus, it could be a variant of *A. flavus*, with a niche ability to thrive in the outbreak-hospital environment.

**IMPORTANCE:**

*Aspergillus flavus* can cause severe infections and hospital outbreaks in immunocompromised individuals. Although lack of isogeneity does not preclude an outbreak, our study underlines the value of microsatellite genotyping in the setting of potential *A. flavus* outbreaks. Microsatellite genotyping documented an isogenic hospital outbreak with an internal source. This provided the “smoking gun” that prompted the rapid allocation of resources for thorough environmental sampling, the results of which guided immediate and relevant cleaning and source control measures. Consequently, we advise that vulnerable patients should be protected from exposure and that genotyping be included early in potential *A. flavus* outbreak investigations. Inspection and sampling are recommended at any site where airborne spores might disperse from. This includes rarely accessed areas where air communication to the hospital ward cannot be disregarded.

## INTRODUCTION

*Aspergillus flavus* is a saprotrophic mold often found in decaying material where it produces readily dispersible conidia (1). It can cause invasive aspergillosis (IA) in neutropenic patients, foremost in regions with arid climates (2, 3). It is the second most important cause of IA after *Aspergillus fumigatus* (4). In general, construction work in and around hospitals, along with contaminated air-supply systems, is the most important risk factor for spreading conidia to patient populations at risk of IA outbreaks (5–8). However, inhabitation and propagation of *A. flavus* inside hospitals have also been reported (9).

In 2017–2019, the incidence of IA due to *A. flavus* increased in children treated for high-risk leukemia at a hematologic cancer center in Copenhagen, Denmark, as previously described (10). The outbreak period coincided with window replacements of the entire ward, which may have caused increased mold exposure. The patients had received amphotericin B (AMB) prophylaxis as per protocol at that time (11), and it was speculated that this regimen selected for *A. flavus* breakthrough infections (10, 12). Outbreak isolates were indeed less susceptible to AMB than *A. fumigatus*, as expected (10, 12). All patients recovered from IA with triazole therapy, supplemented with caspofungin until the target plasma concentration was reached. The primary prophylaxis regimen was changed to posaconazole (13) in 2019 with daily caspofungin during antileukemic vincristine treatment. Cleaning and renovation of the ward were intensified. During 2020–2022, the incidence markedly declined, but three additional cases were still observed in patients not on standard protocols.

*Aspergillus* outbreaks often involve a plethora of different genotypes as air contains multiple unrelated clones. Nosocomial outbreak strains are rarely isogenic when recovered from patient and environmental samples (7, 14, 15). However, isogenic nosocomial outbreaks have been described with direct links to in-ward contamination with *A. flavus* (9, 16–19). Apparent sources have been linked to water damage in plasterboards and other building materials, dust accumulation on acoustical ceiling tiles, defect air-ventilation filters, etc.

To identify the source of this outbreak, we used microsatellite genotyping of patient and air sample isolates. Microsatellite genotyping is an effective tool for investigating the molecular epidemiology of *Aspergillus* isolates (14, 17, 18, 20). We included outbreak isolates and comparative isolates from the rest of the country. We found a single-linage *A. flavus* as the causative agent of this outbreak. A minimum spanning tree (MST) and discriminant analysis of principal components (DAPC) revealed a potentially distinct genetic background of the outbreak clone. All technical risers in the ward were sealed as a consequence of retrieving several isolates from these locations. Repairs of any damaged ward interior, fungal disinfection, and thorough cleaning were done. No *A. flavus* was found in follow-up air samples.

## MATERIALS AND METHODS

### Setting

**Fig 1:**
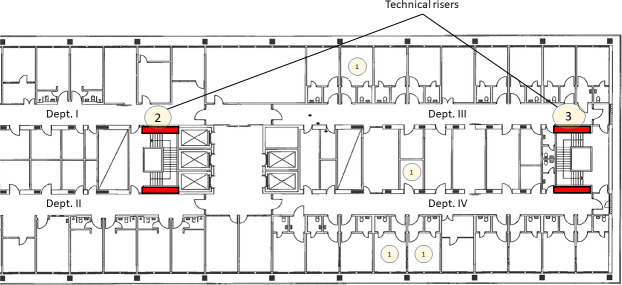
Floorplan of the outbreak ward. *A. flavus* was found in dept. I, III, and IV. The number inside the circles indicates *A. flavus* colony forming units (CFU) found in air samples, in each location. Technical risers (red) are vertical corridors for water piping, running from the basement to the building roof. Unsealed cabinet doors separate them from the wards.

The outbreak hospital is a tertiary referral center with approx. 1,250 beds and 18,000 discharges a year. The outbreak ward is a general pediatric hematologic-oncologic ward, comprising three medical units with a total of 20 beds (Fig. 1), with rooms for medical staff, ventilation, technical risers, etc. separating crosswise (patient rooms face the windows). The ward is not equipped with high-efficiency particulate air filtration. Hematopoietic stem cell transplantations are performed in a different ward.

### Clinical isolates

A total of 140 clinical *A. flavus* isolates were included, including 14 outbreak isolates from eight outbreak ward patients (2018–2022). A total of 126 were clinical comparator isolates from 111 patients admitted to 14 hospitals or referred via general practitioners (1994–2023). Primary patient specimens (PPS) and pure cultures, previously referred for routine diagnostics at the mycology reference laboratory at Statens Serum Institut, were included.

### Control strains

Four external *A. flavus* reference strains were included: three from an external quality-assessment provider, UK-NEQAS (strain 4903, 5246, and 6719, London, UK) and one from Westerdijk Institute (CBS 128202, Utrecht, The Netherlands).

### Environmental sampling

Eighty-six hospital air samples (2 m^3^ per sampling site) were collected with a MAS-100NT air sampler (Merck, Søborg, Denmark), from December 2022 to June 2023. Samples were cultured on YGC-agar at 37°C for 2 days to favor the growth of *Aspergillus*, followed by 3 days at room temperature to facilitate the growth of other fungi for total spore count estimates. Air quality was evaluated using the following categories: areas with critically immunocompromised patients (<1 CFU/m^3^) (21), areas of patients vulnerable to IA (1 ≤ 5 CFU/m^3^—Prof. Malcolm Richardson, personal communication), no particular risk of IA (5 ≤ 25 CFU/m^3^), and unacceptable in general (>25 CFU/m^3^) (21). Identification was based on morphology and MALDI-TOF (Bruker, Roskilde, Denmark) using the MSI spectrum database for molds (22, 23) and when needed (*N* = 20) supplemented with the sequencing of ITS, BTUB, and CMD (24).

### DNA extraction

A. *flavus* colonies were sampled with a cotton swab and suspended in 400 µL of lysis buffer (NucliSENS bioMérieux, Herlev, Denmark). Mechanical lysis was done with 1.4 mm zirconium beads (OPS Diagnostics, Lebanon, NJ, USA) in a VORTEX GENIE 2 (Sigma-Aldrich, Søborg, Denmark) for 10 minutes at 30 Hz. After centrifugation, DNA was extracted with an EMAG for cultures and easyMag for PPSs, (bioMérieux, Herlev, Denmark) and eluted in 100 µL. For PPSs, we used 1 mL sample (BAL *n* = 1, enzymatically dissolved biopsies *n* = 2, and feces *n* = 1).

### Microsatellite typing

With some primer modifications (see the supplemental material), the typing method followed reference 20 with nine microsatellite markers. Forward primers were fluorescently labeled with FAM, HEX, and NED in the 5′ ends. The PCR mix of 25 µL consisted of 0.5 µL of each primer (200 nM), 2.5 µL DNA, 12.5 µL Extract-N-Amp PCR ReadyMix (Sigma-Aldrich, Søborg, Denmark), which contained MgCl_2_, dNTP’s, and DNA-polymerase, and 7 µL DNase/RNase-free water [Thermo Fisher Scientific, Roskilde, Denmark (TFS)].

SimpliAmp thermal cycler (TFS) was used for amplification. The program included denaturation (95°C for 10 min), 30 cycles (95°C for 30 s), annealing (58°C for 30 s), elongation (72°C for 30 s), and incubation (72°C for 10 min). PCR of PPSs required 50 reaction cycles. PCR product (1 µL) was added to 13 µL 6.6% GS500-ROX (TFS) size standard/water solution. A denaturation step (95°C for 3 min) followed by instant cooling to prevent reannealing. SeqStudio Genetic Analyzer (TFS) was used for fragment analysis. Peak-Scanner software enabled analyses of fragment peaks (TFS). For conversion of fragment length (bp) to repeat size (STR), we used genome reference data of *A. flavus* strain CBS 128202 (25). Genetic relations among isolates were visualized with an MST (BIONUMERICS). To detect discriminating patterns among the microsatellite markers (K), a DAPC analysis was run (26) using the R package adegenet 2.1.15 (updated 2020). K was selected based on the Bayesian information criteria (BIC-value) and visualized with a scatter plot. Simpson’s index of diversity (D-value) was used to estimate the probability of collecting identical genotypes from unrelated sources, including all epidemiologically non-related isolates (neither from the same patient nor as part of the outbreak).

### Data analysis

Wilcoxon-test was used for direct comparison of CFU/m^3^ for re-tested (paired) locations. Fisher’s exact test was used to compare the CFU/m^3^ of *A. flavus* found over time.

## RESULTS

### Microsatellite typing

**Fig 2:**
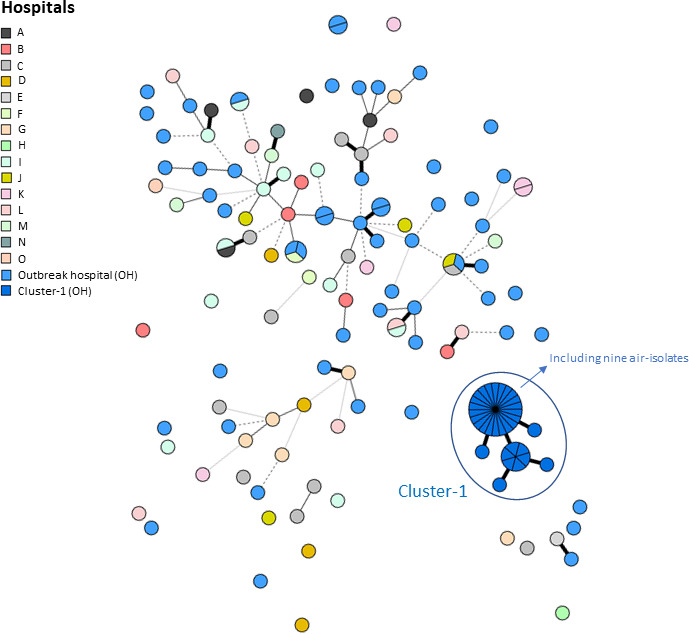
MST of 144 clinical *A. flavus* isolates and nine air-sample isolates. Each node is a multi-locus genotype (MLG), with variable size depending on the number of isolates within. Node distance represents genetic distinction among MLGs. Connecting lines denote a number of varying markers, thick black is a one-marker variation, thin solid is a two-marker variation, dashed is a three-marker variation, etc. Light/dark blue nodes are outbreak hospital (OH) MLGs. Cluster-1 is highlighted.

**Fig 3:**
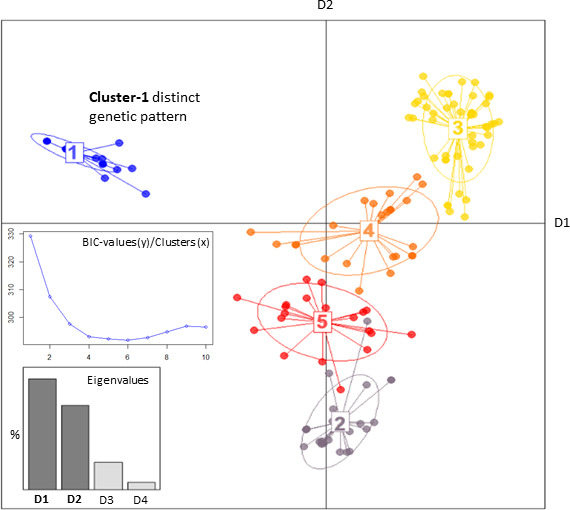
DAPCs, in simple terms, a mathematical model representing the most discriminative differences in the genetic marker patterns, of all isolates in our population. Based on the BIC-value, five clusters credibly show the significant outlying of Cluster-1. Eigenvalues of D1/D2 assure the use of a 2D plot to display almost all discrimination between the clusters (left-hand corner). Clusters 2–5 discriminate mainly along D2, and Cluster-1 discriminate along D1.

Typing of the 140 clinical isolates and four control strains retrieved 116 distinct genotypes (Fig. 2). We calculated a 0.13% probability of detecting identical genotypes among non-related isolates (D-value). One cluster included 23 isolates from 16 different patients, all from the outbreak hospital, and was named “Cluster-1” (Fig. 2 and 3). Genotype and general information on the cluster are shown in Table 1.

**TABLE 1:** Patients in Cluster-1 and the outbreak clone genotype

Cluster-1 patients	Material	Year	2A	2B	2C	3A	3B	3C	4A	4B	4C
Patient 1	Sputum	2008	26	11	12	12	26	9	9	5	9
Patient 2^*a*^^*c*^	Tracheal suction	2018	26	11	12	12	26	9	9	5	9
Patient 3^*a*^^*c*^	Feces	2018	**25** ^*d*^	11	12	12	26	9	9	5	**0** ^*d*^
Patient 4^*a*^^*c*^	BAL	2018	26	11	12	12	26	9	9	5	9
Patient 5	Tracheal suction	2018	**25** ^*d*^	11	12	12	26	9	9	5	9
Patient 6^*b*^	Sputum	2018	26	11	12	12	26	9	9	5	9
Patient 6^*b*^	Sputum	2019	26	11	12	12	26	9	9	5	9
Patient 7^*a*^^*c*^	BAL	2019	**25** ^*d*^	11	12	12	26	9	9	5	9
Patient 8^*b*^	Sputum	2019	26	11	**14** ^*d*^	12	26	9	9	5	9
Patient 9^*b*^	Sputum	2019	26	11	12	12	26	9	9	5	9
Patient 10^*c*^	Biopsy	2020	26	11	12	12	26	9	9	5	9
Patient 10^*c*^	BAL	2020	26	11	12	12	26	9	9	5	9
Patient 10^*c*^	BAL	2020	26	11	12	12	26	9	9	5	9
Patient 10^*c*^	Biopsy	2020	26	11	12	12	26	9	9	5	9
Patient 10^*c*^	Biopsy	2020	26	11	12	12	26	9	9	5	9
Patient 10^*c*^	Biopsy	2020	26	11	12	12	26	9	9	5	9
Patient 11^*b*^	Sputum	2020	26	11	12	12	**27** ^*d*^	9	9	5	9
Patient 12	Spinal fluid	2021	**25** ^*d*^	11	12	12	26	9	9	5	9
Patient 13^*b*^	Sputum	2021	**25** ^*d*^	11	12	12	26	9	9	5	9
Patient 14^*c*^	Spinal fluid	2021	**25** ^*d*^	11	12	12	26	9	9	5	9
Patient 15^*c*^	BAL	2022	**25** ^*d*^	11	12	12	26	**13** ^*d*^	9	5	9
Patient 15^*c*^	BAL	2022	**25** ^*d*^	11	12	12	26	9	9	5	9
Patient 16^*c*^	BAL	2022	26	11	12	12	26	9	9	5	9

^*a*^ First described in 2021 by Vissing et al. as part of an IA outbreak.(10).

^*b*^ Cystic fibrosis patients.

^*c*^ Outbreak ward patients.

^*d*^ Bold font marks deviation from overall genotype.

All Cluster-1 isolates were obtained during 2018–2022 except one from 2008. Eight were derived from outbreak ward patients, six from cystic fibrosis (CF) patients, and for two, information was not available. We typed isolates of 47 additional patients (1994–2023), from other wards of the outbreak hospital, none of whom harbored the Cluster-1 genotype.

### Assessment of indoor air quality

A long-term reservoir within the hospital was suspected on the basis of isogeneity among isolates from the outbreak and the presence of the outbreak clone in the hospital since 2008. As a consequence, air sampling in the outbreak ward was undertaken from December 2022 to January 2023. A total of 258 fungal isolates were cultured from 54 air samples (4.8 CFU/m^3^, range: 0–32.5 CFU/m^3^ per sample). The overall fungal CFU/m^3^ varied 7.0, 5.1, and 3.3, respectively, among the three dept. (I, III and IV) of the ward. A higher *Aspergillus*/total-CFU proportion (39.5%, 17/43 CFU) was found in the technical risers compared to the ward (16.67%, 43/258 CFU). The 43 *Aspergillus* spp. included *A. fumigatus* (17 isolates), *A. flavus* (nine isolates), *Aspergillus calidoustus* (nine isolates), *Aspergillus niger* (six isolates), and *Aspergillus sydowii* (two isolates). Of the nine *A. flavus* isolates found in the air, all had the Cluster-1 genotype. Five of these were found in two of the three technical risers (Fig. 1).

### Genetic diversity and further investigations on the outbreak clone

Genetic diversity of the 153 *A*. *flavus* isolates was visualized in an MST that shows Cluster-1 as a solitary group in distinction to all other clones (Fig. 2). DAPC analysis identified the main discriminative marker patterns and divided the data into clusters, representing these patterns. The Cluster-1 marker pattern clearly discriminated from all others (Fig. 3)

### Infection control and re-assessment of air quality post-intervention

Repairs of any damaged ward interior, fungal disinfection, cleaning of the outbreak ward, and sealing of the technical risers were effectuated by the infection control team. Thirty-two new air samples were collected in areas of dept. I, III, and IV where *A. flavus* or high CFU/m^3^ was found. A total of 104 fungal isolates (3.3 CFU/m^3^) were isolated, none of which were *A. flavus* (*P* = 0.026 comparing 9 *A*. *flavus* among 54 samples with 0 *A*. *flavus* among 32 samples in the two periods, respectively). The overall CFU/m^3^ of fungi for dept. I, III, and IV decreased numerically, from 7.0, 5.1, and 3.3 CFU/m^3^ to 2.5, 3.5, and 3.3 CFU/m^3^, respectively (*P* > 0.5 comparing findings in locations sampled both pre and post intervention). *Aspergillus* spp. included: *A. fumigatus* (14 isolates) and *A. niger* (three isolates).

## DISCUSSION

*Aspergillus* is ubiquitous and thrives in decaying matter. Consequently, construction/renovation work with associated release of fungal spores and contaminated air supply systems remain the most frequent causes of *Aspergillus* outbreaks in hospitals (7). Construction-related outbreaks are most often multi-clonal (14, 15, 18, 27). Thus, we expected limited matches between environmental and clinical isolates in this case as well. However, typing of *A. flavus* from this outbreak revealed a single-lineage outbreak clone in both clinical and environmental air samples. A possible contamination source could therefore be a specific source of decaying building material, possibly exposed to the air in the process of the window replacement.

Others have found decaying building material to be the source of in-hospital *A. flavus* propagation. Examples were moistened insulation material of ductwork, plasterboard, acoustical ceiling tiles, and also dust (9, 16). This was the case in a study from reference 16, which reported an outbreak of IA in a cardiac surgery ward caused by *A. flavus* (16, 28). Air and surface samples were collected, and genetic isogeneity was found in all isolates from environmental and clinical samples. Subsequent to that outbreak, air/surface sampling disclosed an increasing gradient of *A. flavus* isolates toward the focal point of contamination, apparently water damage beneath the ward floor (16). Other examples exist where matching genotypes between clinical and environmental *A. flavus* isolates have been found (15, 17, 19). One study used a similar microsatellite panel to ours and found a cluster of four patients and four environmental isolates from a bronchoscopy ward, where all four patients had been treated (17). These studies support the legitimacy of our findings of a single predominant outbreak clone.

In our study, the earliest outbreak clone isolate was found in a patient in 2008, which indicates the presence of the lineage for more than a decade. Conidia from the outbreak clone may have lingered in the outbreak ward for many years without access to patients. Conidia contained in dust may have contaminated the outbreak ward during the window replacement, thus leading to the outbreak. Conidia possibly settled in areas with no regular cleaning routine, like the technical risers. This is supported by the repeated finding of the outbreak clone in the technical risers and the inability to find it in the post-intervention air samples after the technical risers had been sealed. Technical risers could potentially also contain particularly suitable material for fungal growth, as the risers house hot-water piping offering suitable propagation temperature. Sealing and cleaning of the risers were requested as part of the infection control measurements, in the aftermath of our findings. Unfortunately, this prohibited further sampling of the riser’s interior.

Several studies have affirmed microsatellite typing as an effective tool for discrimination between different lineages of molds within a population (4, 14). Stability, low costs, and high discriminative power among non-related isolates are large benefits. Indeed our calculation of D-value rendered a similarly high discriminative value (0.9987), which corresponds well with other studies using a similar microsatellite panel (17, 20). Furthermore, a high interlaboratory reproducibility gives this method a clear advantage compared to other typing methods (4). The MST (Fig. 2) visually reflected the linkage of Cluster-1 isolates within the *A. flavus* population. The fact that this lineage was found solely in patients from the outbreak hospital and multiple times in the hospital environment gives epidemiological evidence of a single-source nosocomial outbreak. However, DAPC analysis (Fig. 3) revealed the genetic distinction of the outbreak clone genotype compared to all other *A. flavus* isolates. This suggests a different genetic inheritance of the outbreak clone compared to other *A. flavus* clones. This raised the question of whether the outbreak clone had a competitive genetic trait. Of note, no other genotypes of *A. flavus* were found in the hospital air, and this isogeneity does not merely reflect rarity in culturing *A. flavus* from air samples in outbreak settings (9, 15, 29). It may indicate an advantage in adaptability to survive in the hospital environment compared to other *A. flavus* clones. WGS-based typing would be interesting to further elucidate this relationship and to discover if this outbreak clone might even be a subspecies under the *A. flavus* complex.

In conclusion, we learned that one specific lineage of *A. flavus* clones lingers in the outbreak ward, and it has a distinct microsatellite marker pattern compared to other *A. flavus* clones. We speculate whether this has any influence regarding virulence, pathogenicity, or adaptability. However, the outbreak initially described by reference (10) showed no mortality from the outbreak itself. An unsuspected outcome compared to other outbreaks, with high mortality in similar patient populations (7). This might point toward a lower virulence. Air sampling was useful inside the outbreak ward, and we managed to detect the outbreak clone inside the ward itself. Besides genotyping during outbreaks of IA, we emphasize the need for regular cleaning that includes obscure sites like technical risers where contamination of patient environments cannot be excluded due to maintenance. We aim to further monitor the hospital environment and investigate this particular outbreak clone to better understand its nature.

## SUPPLEMENTAL MATERIAL

Supplemental material spectrum.00273-24-s0001.pdf Amplification primers and repeat characteristics.

ASM does not own the copyrights to Supplemental Material that may be linked to, or accessed through, an article. The authors have granted ASM a non-exclusive, world-wide license to publish the Supplemental Material files. Please contact the corresponding author directly for reuse.
