# Supplementary material for: A single-source nosocomial outbreak of Aspergillus flavus uncovered by genotyping
Source: Microbiol Spectr. 2024 Jun 18;12(8):e00273-24. doi: 10.1128/spectrum.00273-24 (PMC11302659; doi:10.1128/spectrum.00273-24)
Supplement: Supplemental material — Amplification primers and repeat characteristics. [file spectrum.00273-24-s0001.pdf]

Supplementary material

Primers Amplification primers and repeat characteristics

PCR panel	Primer sequence (5´-3´) Forward primer	Reverse primer	Repeat unit
M2			
M2A	FAM-TCTCTCTGGGGTGAAGTCTGA*	GTCTGCCTGTACGCCTCTCTT	TG
M2B	HEX-GGTTCTCGAGTCGGTTTGAT*	GAGACCTTTTGCAATCAGCA	GA
M2C	TAMRA-AATCAAGAGCAAGACGTCCA	GTTGAGGCGCTTTCCAACTAC	CA
M3			
M3A	FAM-CATTGCATGTTAGCCCAAAG*	GGTAATCCAGATGCGCTGTT	AAG
M3B	HEX-CCTCGATGGTGAGAGGCTTA	GGGATGATTTCGAGGTCCTT	AAG
M3C	TAMRA-CCAAACATGGCAGAATCAAA*	GTTGAGACGGAGAAGCGAAG*	AAT
M4			
M4A	FAM-GAGGTGTTTGGGTGTCCTTGT	TCATCAAGATACAACACCCAGCTA	TCTT
M4B	HEX-CTGAAAGGGTAAGGGGAAGG	GCAGGGAATACAGCACAACG	TAGG
M4C	TAMRA-CATGAAAAGTATGGCGCAAA	GATGGTTTCTCGCGATTTGT	AAAG

FAM, 6-carboxyfluorecein; HEX, hexachlorofluorecein; TAMRA, tetramethylrhodamine
*Unmodified from Rudramurthy et al. 2011
